# First insights into the rhizospheric bacterial abundance data of *Ceriops tagal* (Perr.) C.B.Rob. from Indian Sundarbans

**DOI:** 10.1016/j.dib.2022.108468

**Published:** 2022-07-14

**Authors:** Gaurab Aditya Dhar, Sayak Ganguli, Bidisha Mallick

**Affiliations:** a Post Graduate Department of Botany, Lady Brabourne College, Kolkata, India; b Post Graduate Department of Biotechnology, St. Xavier's College (Autonomous), Kolkata, 700016, India

**Keywords:** Rhizosphere, Bacterial abundance, Illumina sequencing, Indian Sundarbans

## Abstract

This article reports the analyses of the rhizospheric microbiome of the terrestrial mangrove *Ceriops tagal* (Perr.) C.B.Rob. from the Indian Sundarbans. Samples were collected using standard protocols and 16S rRNA gene V3–V4 region amplicon sequencing was performed to identify the bacterial communities prevalent in the rhizosphere. A total of 1,74,324 quality checked reads were assembled into contigs and were analyzed using QIIME2 and MetaG server to reveal the abundance of *Proteobacteria, Bacteroidetes* and *Actinobacteria*. The data is available at the NCBI - Sequence Read Archive with accession number: SRR15652592. This is the first report of the rhizospheric microbiome belonging to this plant from the Indian Sundarbans.

## Specifications Table

**Table:** 

Subject	Biological Sciences
Specific subject area	Rhizobiome Bacterial Profiling
Type of data	NGS Based Data represented in the form of Pie Chart
How the data were acquired	Illumina HiSeq Next Gen Sequencing Platform; FASTQC; QIIME2; MetaG
Data format	Raw
Description of data collection	DNA isolation and sequencing from bacterial population in the soil obtained from plant rhizosphere
Data source location	• *Institution:* St. Xavier's College (Autonomous), Kolkata and Lady Brabourne College, Kolkata • *City/Town/Region:* Kolkata • *Country:* India • *Latitude and longitude (and GPS coordinates, if possible) for collected samples/data:* Dobanki, Sunderban [22.16°N 88.80°E]
Data accessibility	Repository name: NCBI – Sequence Read Archive Data identification number: SRR15652592 Direct URL to data: https://www.ncbi.nlm.nih.gov/sra/?term=SRR15652592

## Value of the Data

• The importance of this data lies in that it is a data amounting to the first report of bacterial abundance using 16S rRNA sequencing using NGS platforms in the rhizosphere of *Ceriops tagal* (Perr.) C.B.Rob. from the Indian Sundarbans, which can be used for future comparative metagenomic analyses.
• *Ceriops tagal* (Perr.) C.B.Rob. is one of the most important true mangrove species of the Indian Sundarbans which is fast declining from populated islands. Thus, a proper idea in terms of it's rhizospheric bacterial assemblage would help us to analyse the factors that are contributing towards it's habitat loss.
• The data is valuable for the purposes of ecological monitoring of the Indian Sundarbans which is currently under the onslaught of increasing cyclonic activities and salinity ingression into agricultural land [1].
• The data will enhance and augment ongoing mangrove restoration approaches and practices [2] which will help to curb accelerated loss of deltaic soil in the Indian Sundarbans and will benefit the indigenous human population and fauna by assisting fertile land reclamation and land recovery.

## 1 Data Description

The paired end reads uploaded on the Nephele server returned a complete QC analysis of the data. The QC statistics are listed in the Table 1.

**Table 1:** Summary Statistics of FASTQC analysis.

Sample Name	% Dups	% GC	Length	% Failed	Seqs
Ceriops_Rhizo_L1_R1	96.1%	54%	250 bp	33%	174406
Ceriops_Rhizo_L1_R2	94.3%	52%	250 bp	33%	174406

MetaG server data outputs were classified according to the taxonomic ranks of the OTU clusters. Of the 1,74,406 OTUs, 82 failed to pass the QC filter and 1,74,324 OTUs were matched to reference, all of which were found to be of bacterial origin, explaining the nature of dominant domain composition of the rhizospheric soil sample of *Ceriops tagal* (Perr.) C.B.Rob. The alpha-diversity of this data set was identified to be 9.181, with the Shannon diversity index value of 4.298 and Faith's Phylogenetic Diversity (PD) index value at 8.380.

The phylum level data exhibits the highest abundance of *Proteobacteria* (79.8%), followed by *Bacteroidetes* (12.5%), *Actinobacteria* (4.9%), *Firmicutes* (2.05%), *Cyanobacteria* (0.46%), *Planctomycetes* (0.15%) and *Chloroflexi* (0.04%) respectively with unclassified bacterial sequences amounting to 0.16%. Of the phylum *Proteobacteria, Gammaproteobacteria* (49.3%) and *Alphaproteobacteria* (12.5%) was found to contribute majorly to the class level abundance. *Deltaproteobacteria* (10%) and *Betapropteobacteria* (7.85%) also ranked in majority only to be flanked by *Flavobacteria* (11.9%). Class *Actinobacteria* (4.9%) and *Bacilli* (1.1%) was found to follow the preceding members.

Order *Oceanospirillales* (22.6%) of the class *Gammaproteobacteria* was found to be most abundant, followed by *Pseudomonadales* (20.5%), *Flavobacteriales* (11.9%), *Bdellovibrionales* (9.8%), and *Caulobacterales* (8.05%).

**Fig. 1:**
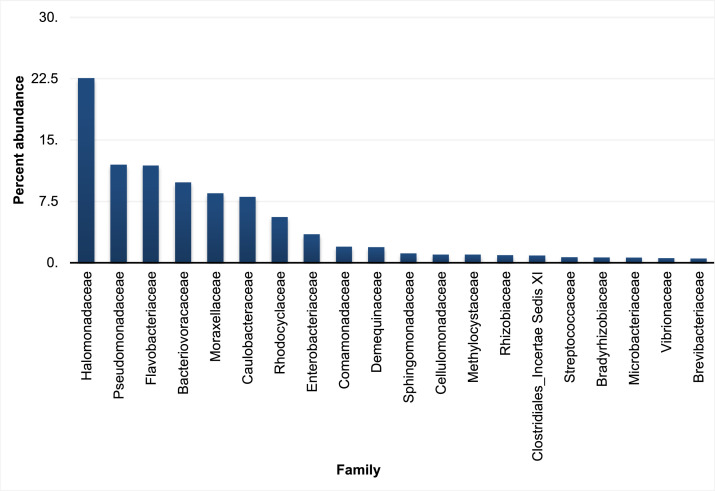
Top 20 families (except unclassified) according to percent abundance of the rhizobial assemblage of *Ceriops tagal* (Perr.) C.B.Rob.

The family level data of the sample exhibited the presence of *Halomonadaceae* (22.6%) as the major constituent of all the family of bacteria present. *Pseudomonadaceae* (11.96%), *Flavobacteriaceae* (11.89%), *Bacteriovoracaceae* (9.82%), *Moraxellaceae* (8.49%), *Caulobacteraceae* (8.05%) and *Rhodocyclaceae* (5.58%) respectively contribute significantly to the family level abundance data along with others. The Family-level abundance is represented in the bar graph as Fig. 1.

**Fig. 2:**
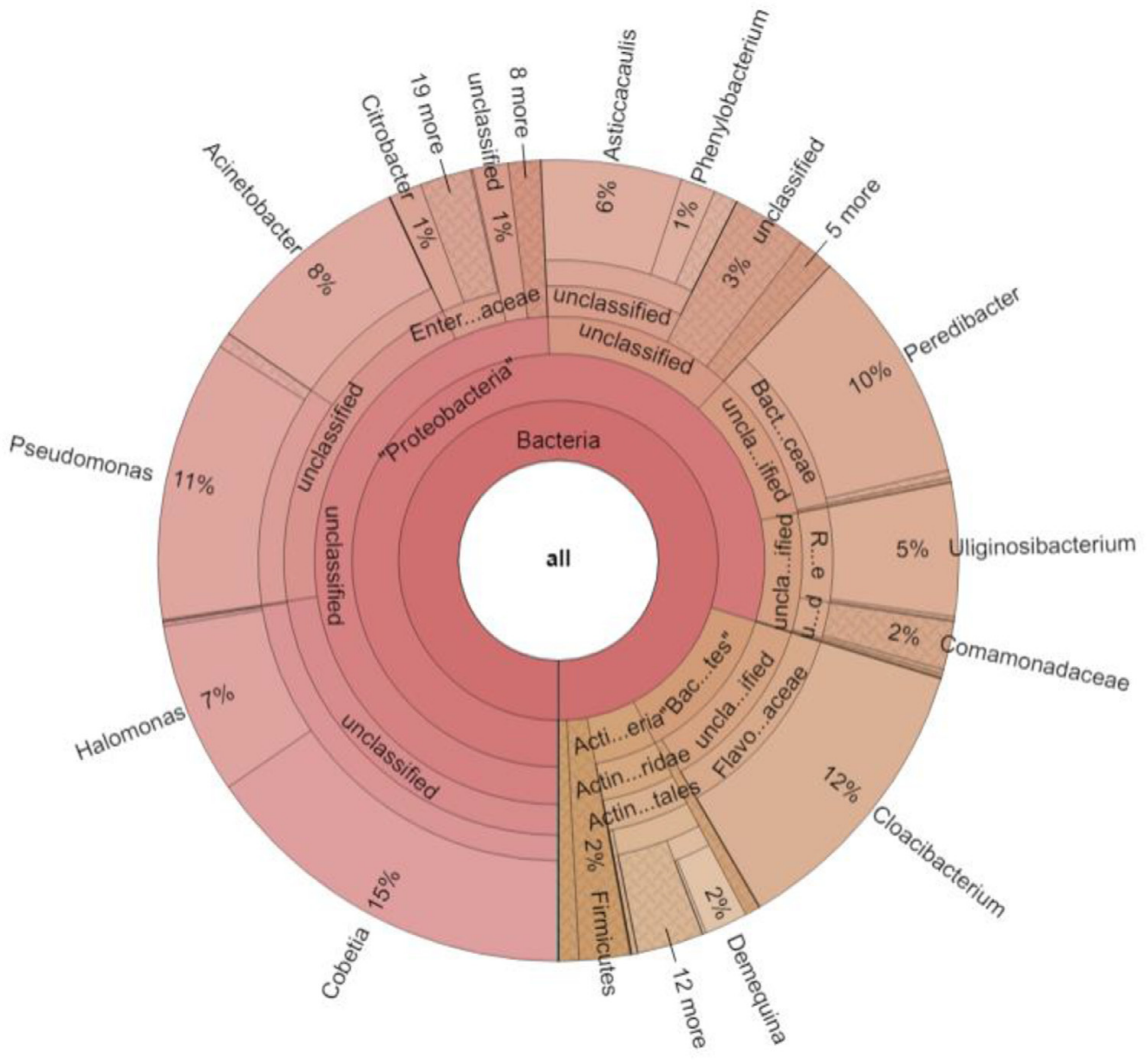
Genus-level abundance of the rhizospheric assemblage of *Ceriops tagal* (Perr.) C.B.Rob.

At the Genus level, *Cobetia* (15.45%) appeared to be the most dominant one, closely followed by *Cloacibacterium* (11.86%) and *Pseudomonas* (11.35%). *Peredibacter* (9.53%), *Acinetobacter* (8.47%), *Halomonas* (6.95%), and *Asticcacaulis* (5.68%) were the other genera that follow. The Genus-level abundance is represented in the Krona chart as Fig. 2.

## 2 Experimental Design, Materials and Methods

### 2.1 Rhizospheric soil collection and metagenomic sequencing

The 16S rRNA gene consists of nine hypervariable regions interspersed between conserved regions, which has found widespread usage in identifying and characterizing the bacterial community of an environmental sample. In the present analysis, microbial community structure was identified by targeting V3–V4 region, as these regions show high variability which helps to distinguish bacterial subtypes.

### 2.2 Collection of sample

Maximum population density of *Ceriops tagal* (Perr.) C.B.Rob. at a specific site was used to determine the sampling site. The soil was deltaic alluvium with a slightly acidic pH and heavy-textured with a mixture of silt, sand, clay and mud. Rhizospheric soil sample was collected from an appropriate depth beneath the muddy swamp base that is less prone to the semi-diurnal tidal inundation. Sample collection was performed wearing gloves and mask to reduce any chance of anthropogenic contamination. The soil sample was placed in a sterile zip lock packet and kept in an ice box at 4°C for preservation.

### 2.3 Preparation of sample

An in-house standardized protocol was used for isolation of genomic DNA from a rhizospheric sample. DNA quality was assessed by Nanodrop and on agarose gel, followed by quantification using QUBIT. The library preparation was carried out using Illumina standardized V3-V4 regions of the 16S rRNA library protocol. The enriched library was quantified and validated using qPCR and Agilent Bioanalyzer (DNA 1000 chip). The library generated containing V3-V4 amplicons was sequenced on Illumina MiSeq using 300 × 2 PE chemistry.

### 2.4 Bioinformatic analyses

The Bioinformatics pipeline previously reported in [3] was used to analyze the data. The quality control of raw reads was carried out using the FASTQC toolkit (http://www.bioinformatics.babraham.ac.uk/projects/fastqc) via the Nephele: Microbiome Analysis site (https://nephele.niaid.nih.gov/). The quality processed paired end reads were clustered into OTU's (Operational Taxonomic Units) by using QIIME2 software to identify the microbial community.

The processed reads were uploaded to MetaG server (http://www.bioinformatics.uni-muenster.de/tools/metag/index.hbi) for analysis and classification of abundant bacteria to the classification level of Genus and Krona graphs [4] were generated for visualization.

## Ethics Statements

Not applicable.

## CRediT Author Statement

GAD and SG performed the sample collection and processing. SG and BM conceptualized the methodology workflow and GAD performed the analyses. SG and BM were involved in overall supervision of the work.

## Declaration of Competing Interest

The authors declare that they have no known competing financial interests or personal relationships that could have appeared to influence the work reported in this paper.

## Data Availability

Ceriops Tagal Rhizosphere (Original data) (NCBI - SRA).
